# Priorities for vocabulary intervention design using texting — Data to examine the critical role of language learners׳ behaviors and perceptions

**DOI:** 10.1016/j.dib.2018.07.067

**Published:** 2018-07-31

**Authors:** Jia Li, Qizhen Deng

**Affiliations:** University of Ontario Institute of Technology, Boise State University, Canada

## Abstract

We examined the role of university English language learners’ (ELLs) behavior and perception of a texting-based instruction intervention on their academic vocabulary acquisition. This article reports on the data pertaining to 108^1^ ELLs from six undergraduate classes taking two comparable undergraduate courses on content-based English for Academic Purpose (EAP). The data include (1) the performance of the control and intervention groups on pre- and post-intervention tests on target vocabulary and academic vocabulary, (2) a pre-intervention survey of participants’ technology use, and (3) a post-intervention survey of participants on learning behavior during the intervention and their perception of the intervention. Data presented here are related to the article [4].

**Specifications Table**

**Table:** 

Subject area	*Education*
More specific subject area	*Computer assisted language learning, texting, instructional design/intervention, vocabulary acquisition*
Type of data	*Excel files*
How data was acquired	*Surveys; Performance indicators of students from two vocabulary pre- and post-tests*
Data format	*Raw*
Experimental factors	*Data are from a pretest-posttest quasi-experimental design*
Experimental features	*Data includes two pretest scores, and two posttest scores*
Data source location	*Toronto, Canada*
Data accessibility	*Data is accessible through Appendix A at*https://data.mendeley.com/datasets/fn4vmn7chk/2
Related research article	Li and Deng [4]

**Value of the data**

• Data can be further statistically analyzed to examine the profiles of English language learners’ technology use as well as their performance on academic vocabulary.
• Data can be used as a benchmark to iteratively develop texting-based interventions and compare its effect using similar vocabulary performance.
• Data can also be used to identify the appropriate levels of target vocabulary for the texting-based interventions tailored to English language learners with different academic vocabulary proficiency levels.
• Academic vocabulary data for this sample can be compared with that for other samples for further insight.

## 1 Data

The data file (Appendix A) contains nominal, ordinal and continuous variables separated in four sheets including the results from pre- and post-target vocabulary and academic vocabulary tests, pre-intervention survey, and post-intervention survey. The former two spreadsheets consist of several variables (show in column) such as pseudo student ID, Teacher ID, Class ID, intervention condition, time of tests, and the dependent variables target vocabulary and academic vocabulary scores. The latter two spreadsheets include the results from the pre-intervention survey on students’ technology use as well as the results from the post-intervention survey on the intervention group׳s perception of the intervention and learning behavior during the intervention.

## 2 Experimental design, materials and methods

The data for this article was from a larger “Word Matters” intervention study with 108 university English language learners (ELLs) in a large Canadian university. The permission to conduct the study was granted by the Research Ethics Board (REB) of University of Ontario Institute of Technology of the first author׳s affiliation, and the REB of the university where data was collected. Ethics protocols were followed, including a pseudonym and code assigned to each participant.

This research project examined the effect of texting on academic vocabulary learning for ELLs. Data presented here were collected from (1) a pre-intervention survey of students’ background information and their technology use, (2) pre- and post-intervention test scores for all participating students’ performance on target vocabulary and academic vocabulary, and (3) a post-intervention survey of students reporting on their perceptions of the intervention and learning behaviors during the intervention. The target vocabulary test included 60 target words randomly selected from the target vocabulary pool, consisting of 200 academic and low frequency words [2]. The academic vocabulary test was 30-item in the Vocabulary Levels Test (Version 2) [5].

The pre-intervention survey was conducted immediately before the intervention began. The survey included two parts. Part one focused on demographic information such as grade level, first language, country of origin, and years of English instruction. Part two involved six items on a 5-point frequency count scale (i.e., more than once a day, once a day, once a week, once a month, never) asking the frequency of using text messages, emails, Facebook, WeChat, Twitter, and other social media, respectively.

The intervention “Word Matters” aimed to teach university ELLs 189 target words, out of the vocabulary pool, necessary to understand assigned course readings using text messages. The intervention lasted for nine weeks. The target words were carefully selected from the required reading materials for English for EAP classes. Three classes were randomly assigned to the intervention group (*n* = 48) and the other three to the control group (*n* = 60). The treatment included sending ELLs three words daily through texting: one in the morning, one at noon, and one in the afternoon, congruent with the optimal time for learning [1], [3]. Each text message involved a target word, its part of speech, the page reference in the assigned reading, word definition and an example sentence. Word definitions were adapted from classic dictionaries: 126 words from the American Heritage ® Dictionary of the English language (4^th^ edition), 62 words from the Merriam-Webster Learner׳s Dictionary, 8 words from the Merriam-Webster Online Dictionary, 2 words from the Century Dictionary and Cyclopedia, and 2 words from the Collins COBUILD Advanced Learners’ English Dictionary. Example sentences were either from the original textbooks (25 words) or dictionaries, including Merriam-Webster Learner׳s Dictionary (131 words), Collins COBUILD Advanced Learner׳s English Dictionary (32 words), American Heritage Dictionary of the English language (12 words). Through email, students also received a summary of the words learned that day and a quiz on the words learned previously. At the end of each week and each month, a summary of target vocabulary was sent to the students for their own future reference. Participants in the intervention group received a total of 189 target words through text messages, with three words sent daily, or 21 words sent weekly.

The post-intervention survey was conducted immediately after the completion of the intervention. The survey included 11 items on students’ behavior and perception of the intervention. Specifically, the items focused on students’ frequency of reading text messages and emails, perceptions of the easiness of the text content, preferred number of example sentences, number of words per day, and perceptions of the helpfulness of the intervention. A 7-point frequency count scale was used for Item 1 (frequency of reading text messages) and Item 2 (frequency of reading emails). A 5-point Likert-type scale was adapted to measure students’ perception of the difficulty levels of the texting contents (i.e., Item 3, Item 4, Item 5, Item 6) and the helpfulness of the intervention (i.e., Item 10, Item 11). A 5-point frequency count scale was used to examine students’ preferred number of example sentences (Item 7) and number of words daily (Item 8). Item 9 on a 5-point Likert-type scale was used to examine students’ perception of the interest level for the word games/quizzes. See Table 1 in Li and Deng [4] for the details of the frequency count scale and Likert-type scale in the post-intervention survey.

## 3 Note

This number is the maximal number of participants by control and intervention crosstabulation for target vocabulary performance.
