# Supplementary material for: Priorities for vocabulary intervention design using texting — Data to examine the critical role of language learners׳ behaviors and perceptions
Source: Data Brief. 2018 Jul 31;20:200–3. doi: 10.1016/j.dib.2018.07.067 (PMC6097277; doi:10.1016/j.dib.2018.07.067)
Supplement: Supplementary file 1 — Supplementary material [file mmc1.docx]

Conflict of Interest Form

We wish to confirm that there are no known conflicts of interest associated with this manuscript “Priorities for Vocabulary Intervention Design using Texting: Data to Examine the Critical Role of Language Learners' Behaviors and Perceptions”.

There has been no financial support for this work that could have influenced its outcome. We confirm that the manuscript has been read and approved by all named authors. We further confirm that the order of authors listed in the manuscript has been approved by all of us.

We understand that the Corresponding Author is the sole contact for the Editorial process (including Editorial Manager and direct communications with the office).

She is responsible for communicating with the other authors about progress, submissions of revisions and final approval of proofs. We confirm that we have provided a current, correct email address which is accessible by the Corresponding Author.

Corresponding Author:

Jia Li PhD.

Associate Professor

Faculty of Education

University of Ontario Institute of Technology

EDU 521, 11 Simcoe Street North, P.O. Box 385

Oshawa, Ontario, Canada L1H 7R7

Tel: 905.721.8668 EXT.3823

Fax: 905.721.1707

Email: [jia.li@uoit.ca](mailto:jia.li@uoit.ca)

Second Author:

Qizhen Deng, Ph.D.

Assistant Professor

Department of Literacy, Language and Culture

College of Education

Boise State University

[qizhendeng@boisestate.edu](mailto:qizhendeng@boisestate.edu)
